# Supplementary figures and images for: The genetic architecture of helminth-specific immune responses in a wild population of Soay sheep (Ovis aries)
Source: PLoS Genet. 2019 Nov 7;15(11):e1008461. doi: 10.1371/journal.pgen.1008461 (PMC6863570; doi:10.1371/journal.pgen.1008461)

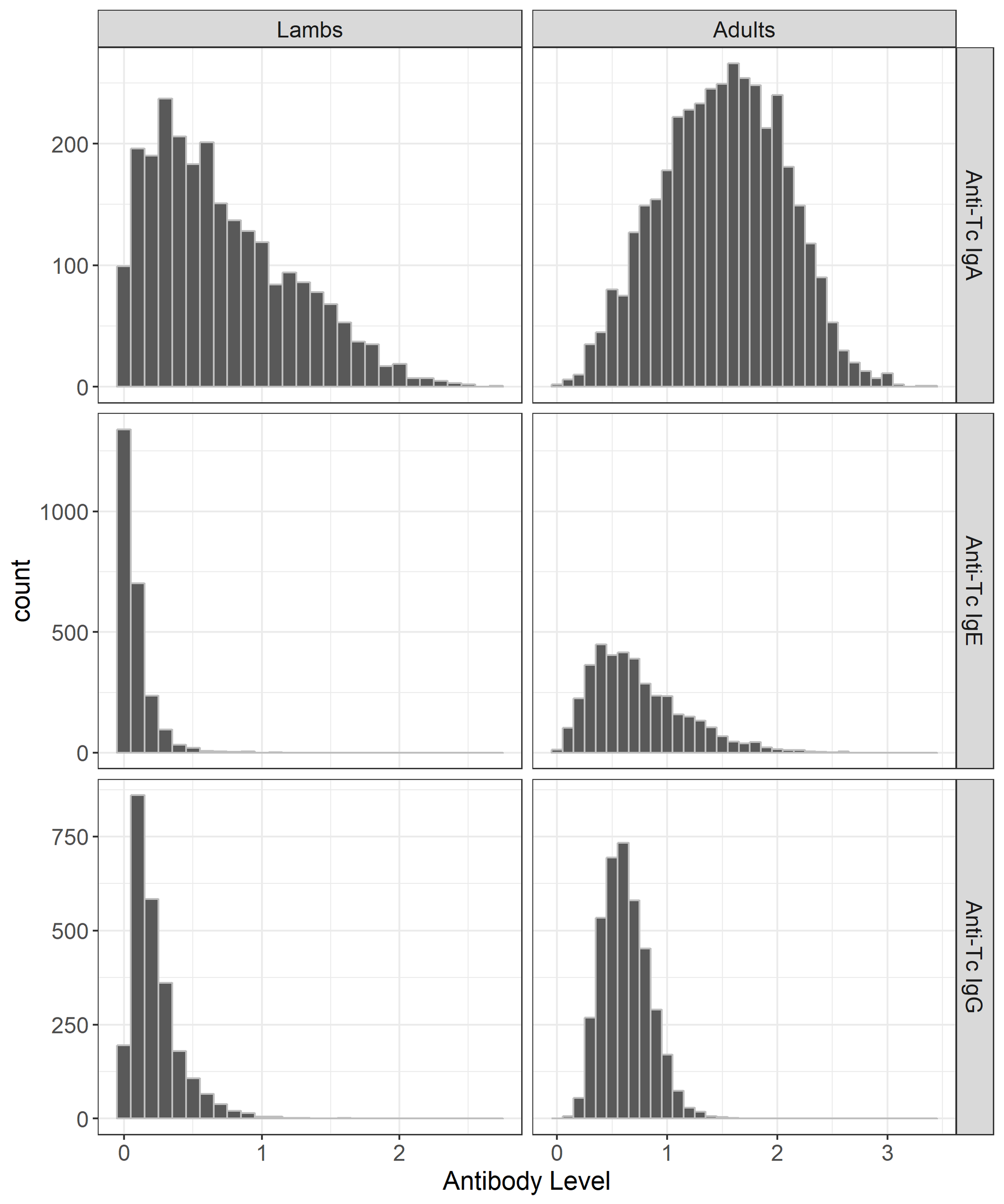

Supplement: S1 Fig — Histograms of anti-Teladorsagia circumcincta IgA, IgE and IgG levels in lamb (left column) and adult (right column) Soay sheep. (TIF) [file pgen.1008461.s001.tif]

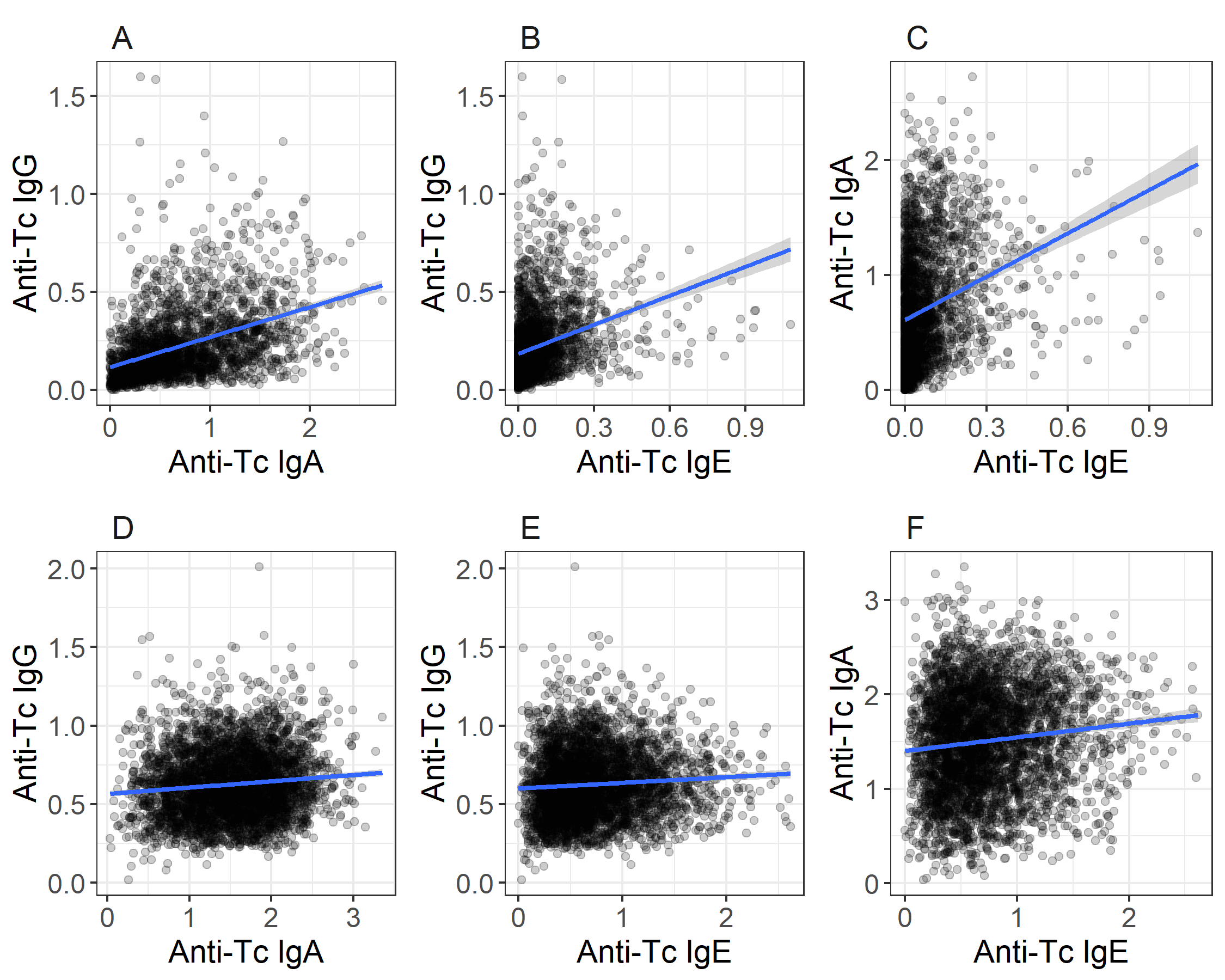

Supplement: S2 Fig — Correlations between anti-T. circumcincta IgG, IgA, and IgE levels in lamb (A-C) and adult (D-F) Soay sheep. Model results are provided in S1 Table. (TIF) [file pgen.1008461.s002.tif]

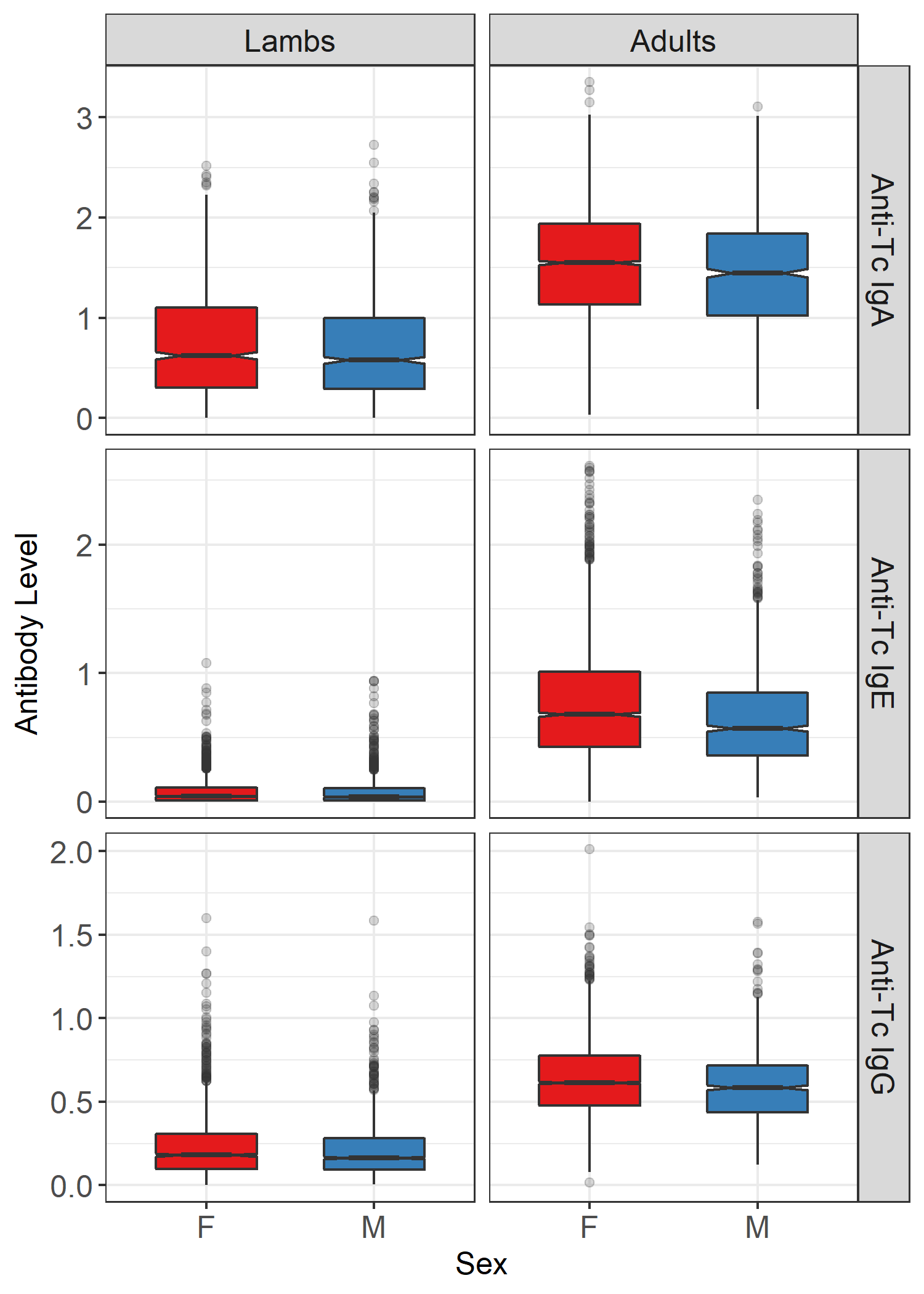

Supplement: S3 Fig — (TIF) [file pgen.1008461.s003.tif]

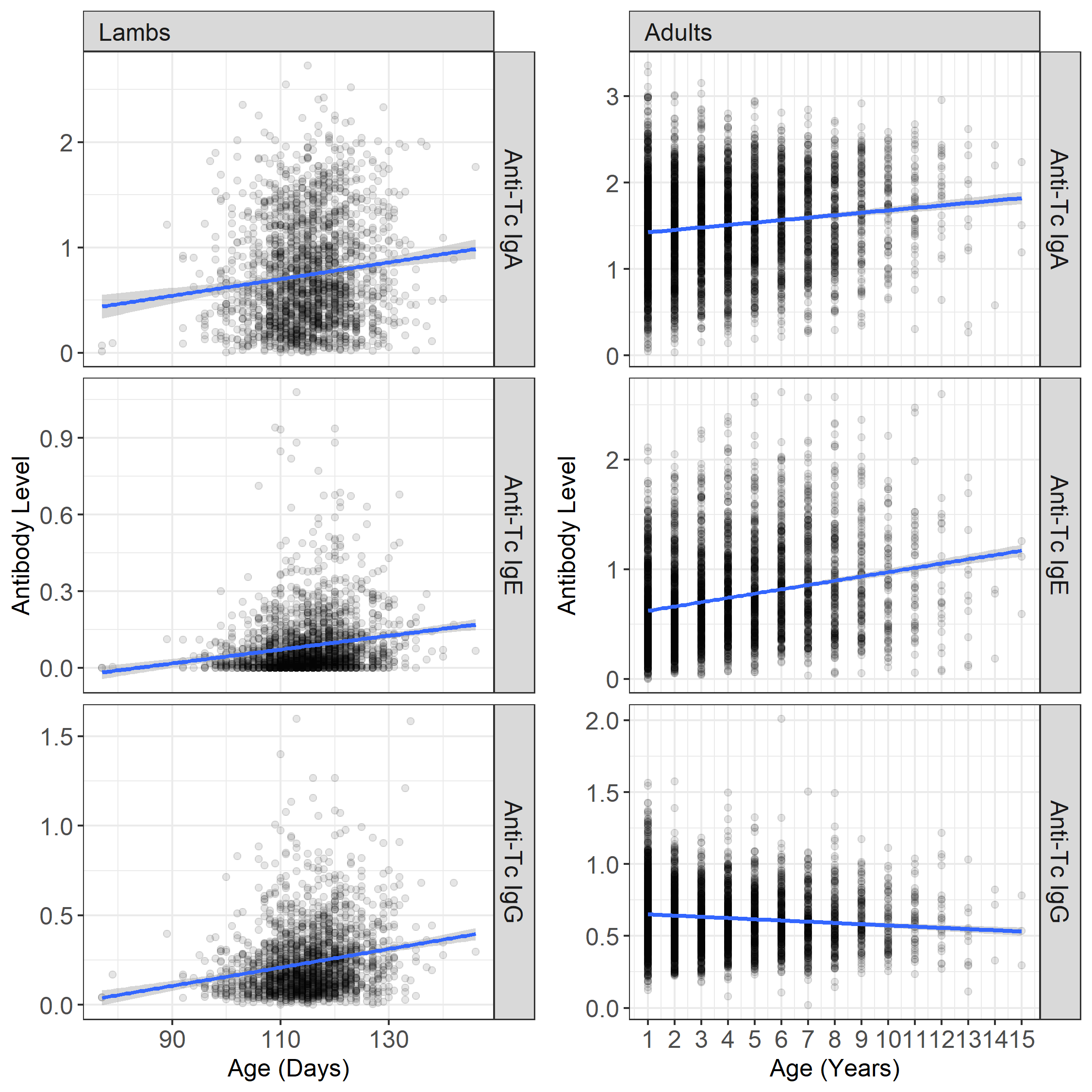

Supplement: S4 Fig — Anti-T. circumcincta IgG, IgA, and IgE levels in lambs with age in days (left) and in adults with age in years (right). Animal model results are provided in S2 Table. (TIF) [file pgen.1008461.s004.tif]

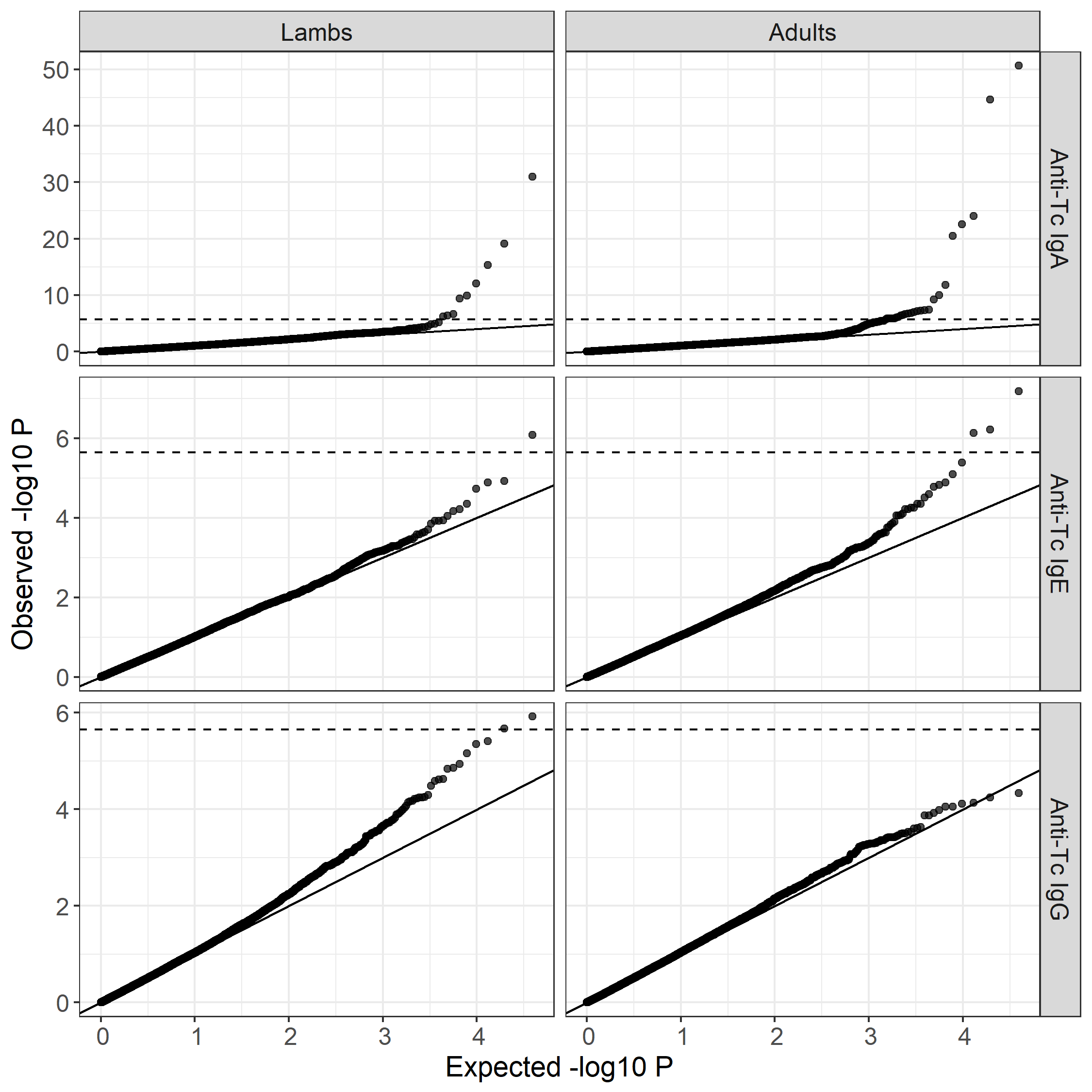

Supplement: S5 Fig — The dotted line indicates the genome-wide significance threshold, and the solid line indicates a 1:1 correspondence between the observed and expected values. (TIF) [file pgen.1008461.s005.tif]

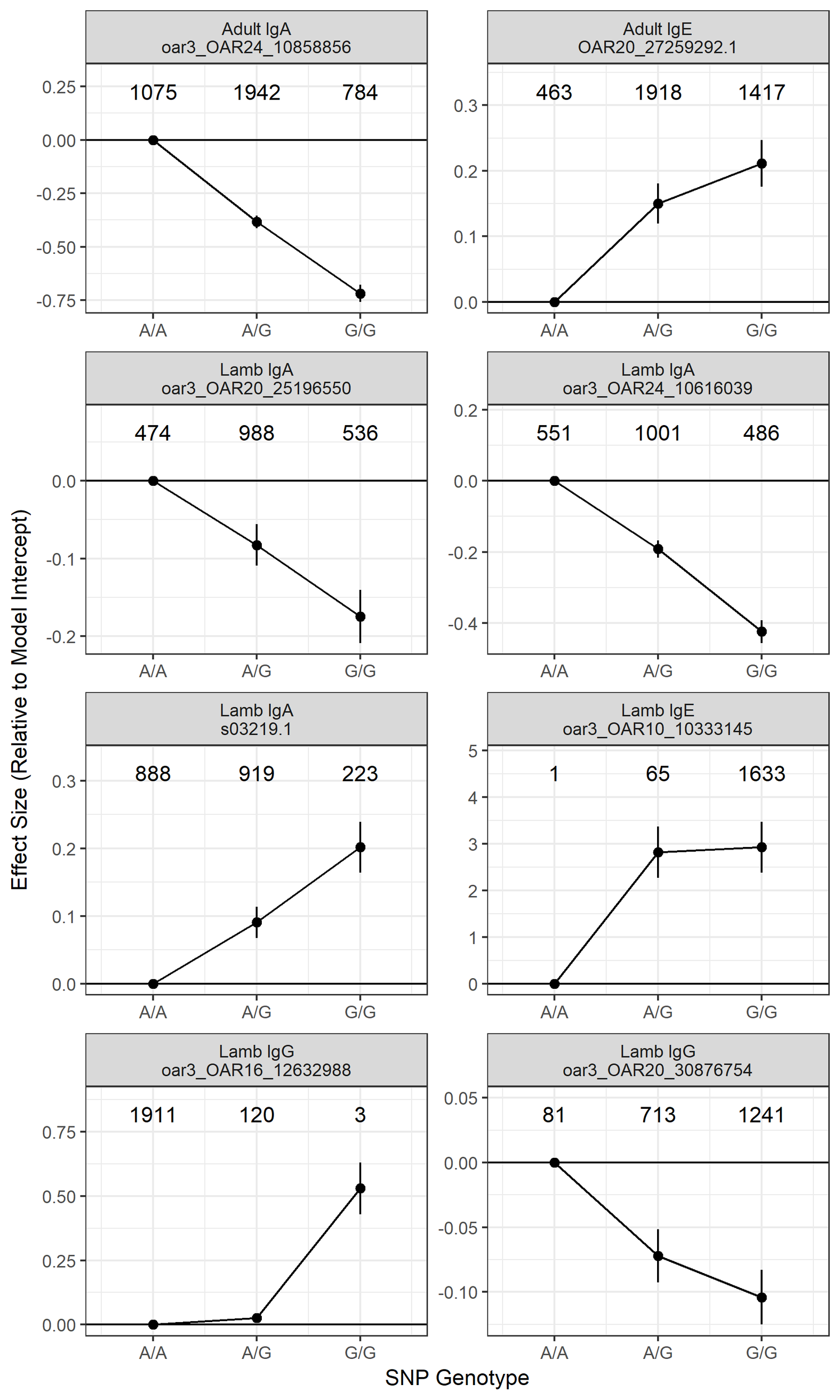

Supplement: S6 Fig — The model intercept is for genotype A/A. The full model results are provided in S9 Table. Sample sizes are provided above each set of points. (TIF) [file pgen.1008461.s006.tif]

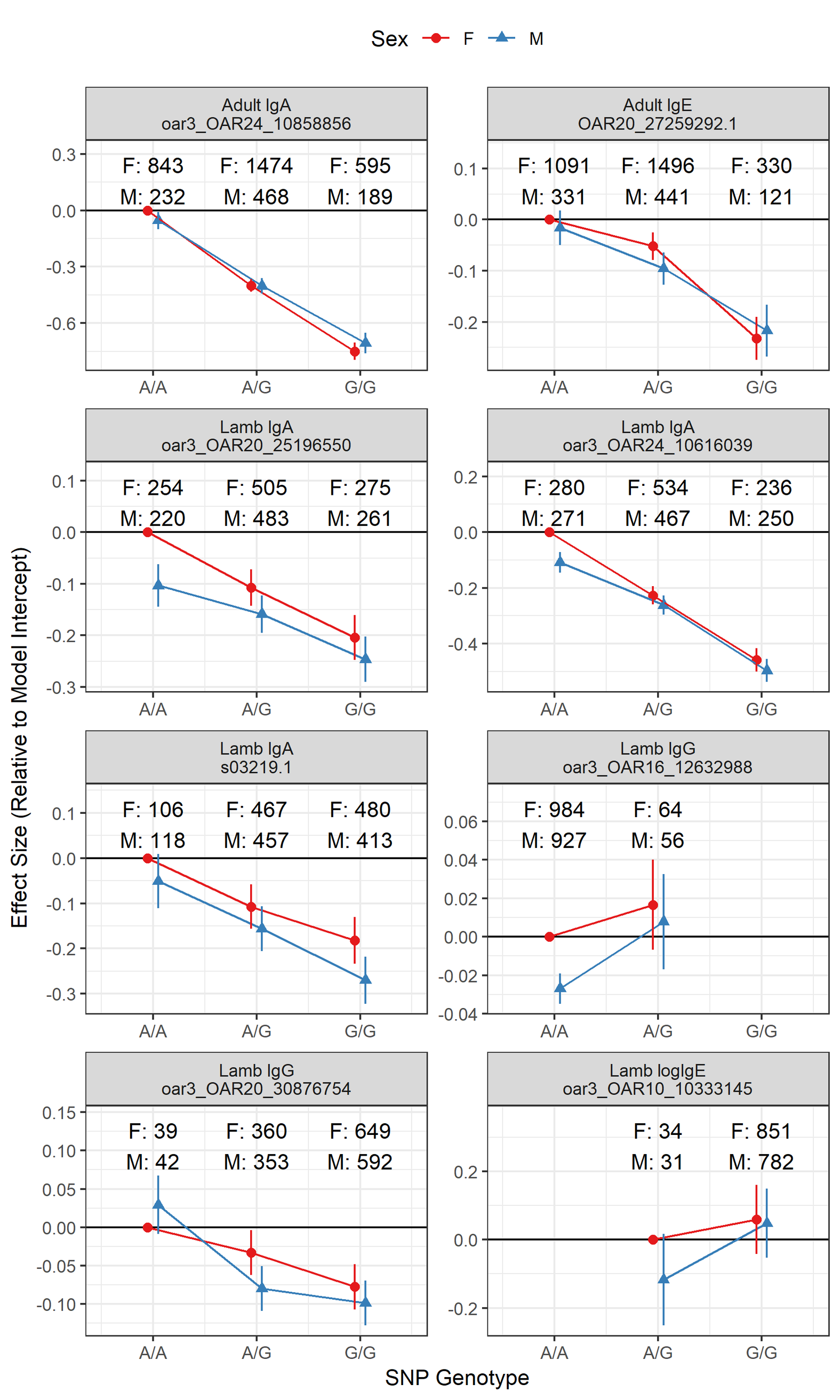

Supplement: S7 Fig — Estimates of genotype effects in females (circles) and males (triangles) in animal models containing a SNP genotype by sex interaction for the most highly associated SNPs in Table 2. The model intercept is A/A females, except for locus oar3_OAR10_10333145, which is A/G females. The model results are provided in S10 Table. Sample sizes are provided above each set of points. (TIF) [file pgen.1008461.s007.tif]

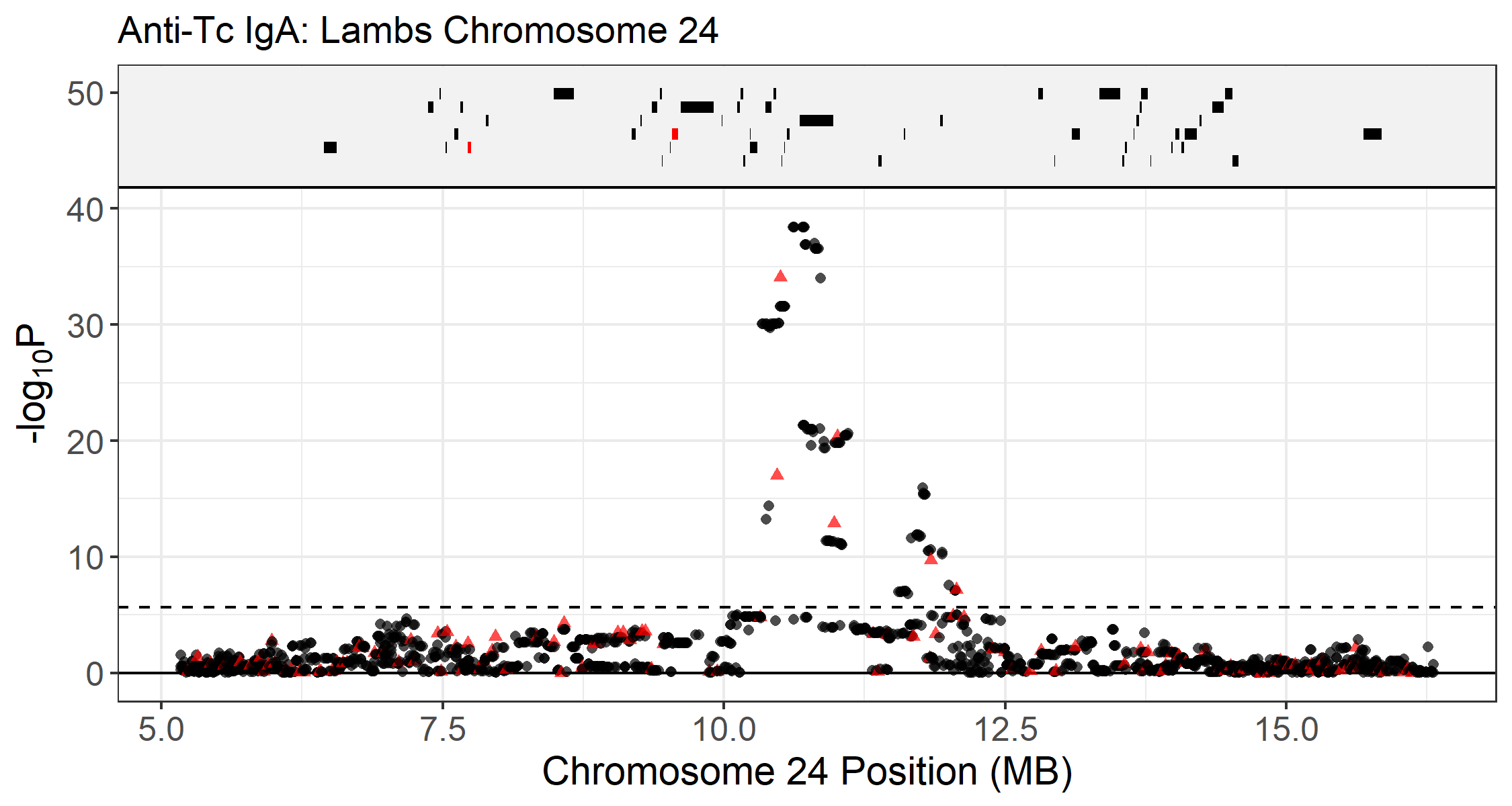

Supplement: S8 Fig — The dotted line indicates the genome-wide significance threshold equivalent to an experiment-wide threshold of P = 0.05. Points are colour-coded by their imputation status i.e. from the SNP50 chip (red triangles) or imputed from the Ovine HD chip (black points). Underlying data, sample sizes and effect sizes are provided in S6 Table. Gene positions are shown in the grey panel at the top of each plot and were obtained from Ensembl (gene build ID Oar_v3.1.94) and are provided in S7 Table. Genes coloured red have GO terms associated with immune traits (S8 Table). (TIF) [file pgen.1008461.s008.tif]

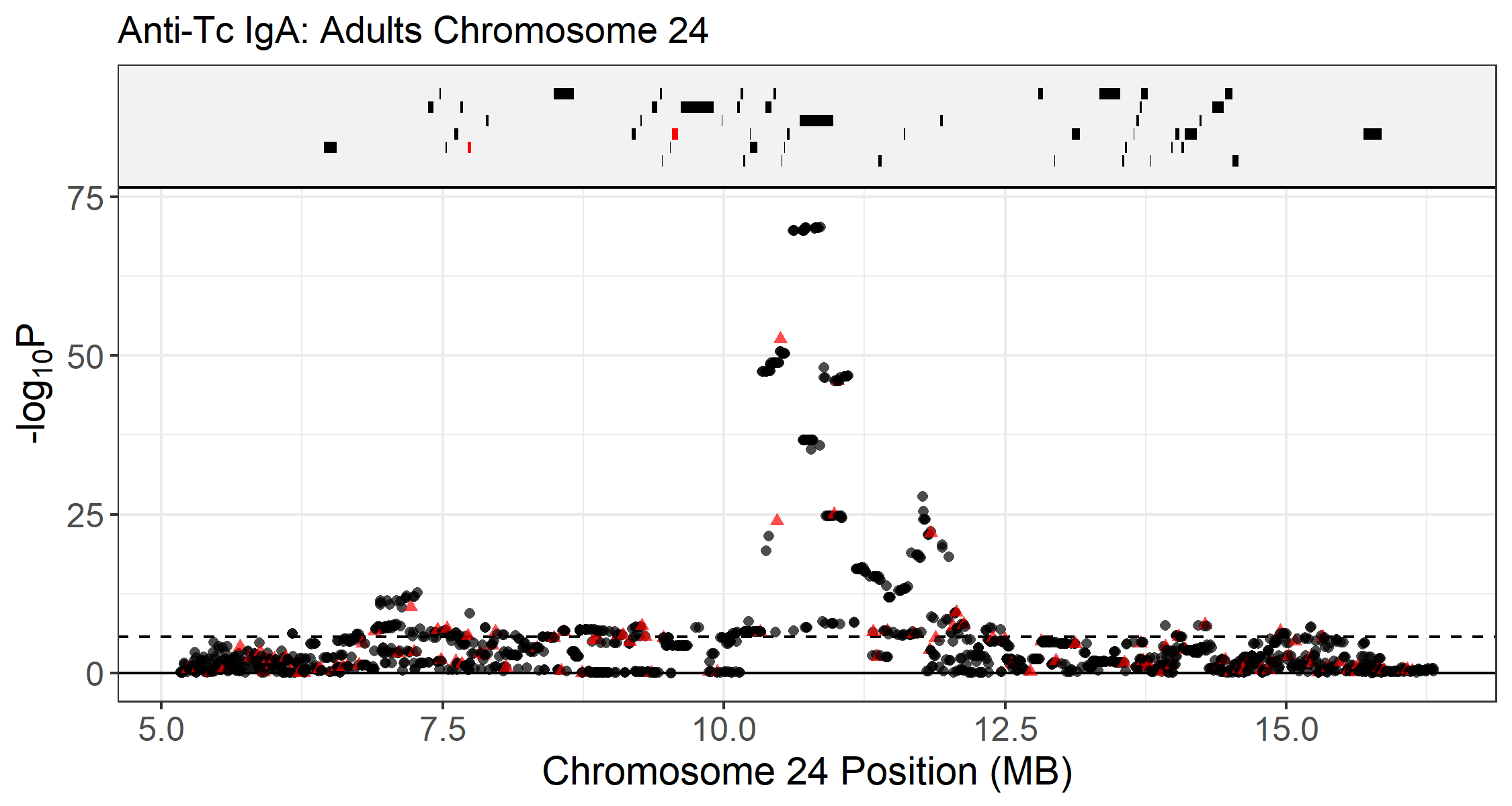

Supplement: S9 Fig — The dotted line indicates the genome-wide significance threshold equivalent to an experiment-wide threshold of P = 0.05. Points are colour-coded by their imputation status i.e. from the SNP50 chip (red triangles) or imputed from the Ovine HD chip (black points). Underlying data, sample sizes and effect sizes are provided in S6 Table. Gene positions are shown in the grey panel at the top of each plot and were obtained from Ensembl (gene build ID Oar_v3.1.94) and are provided in S7 Table. Genes coloured red have GO terms associated with immune traits (S8 Table). (TIF) [file pgen.1008461.s009.tif]

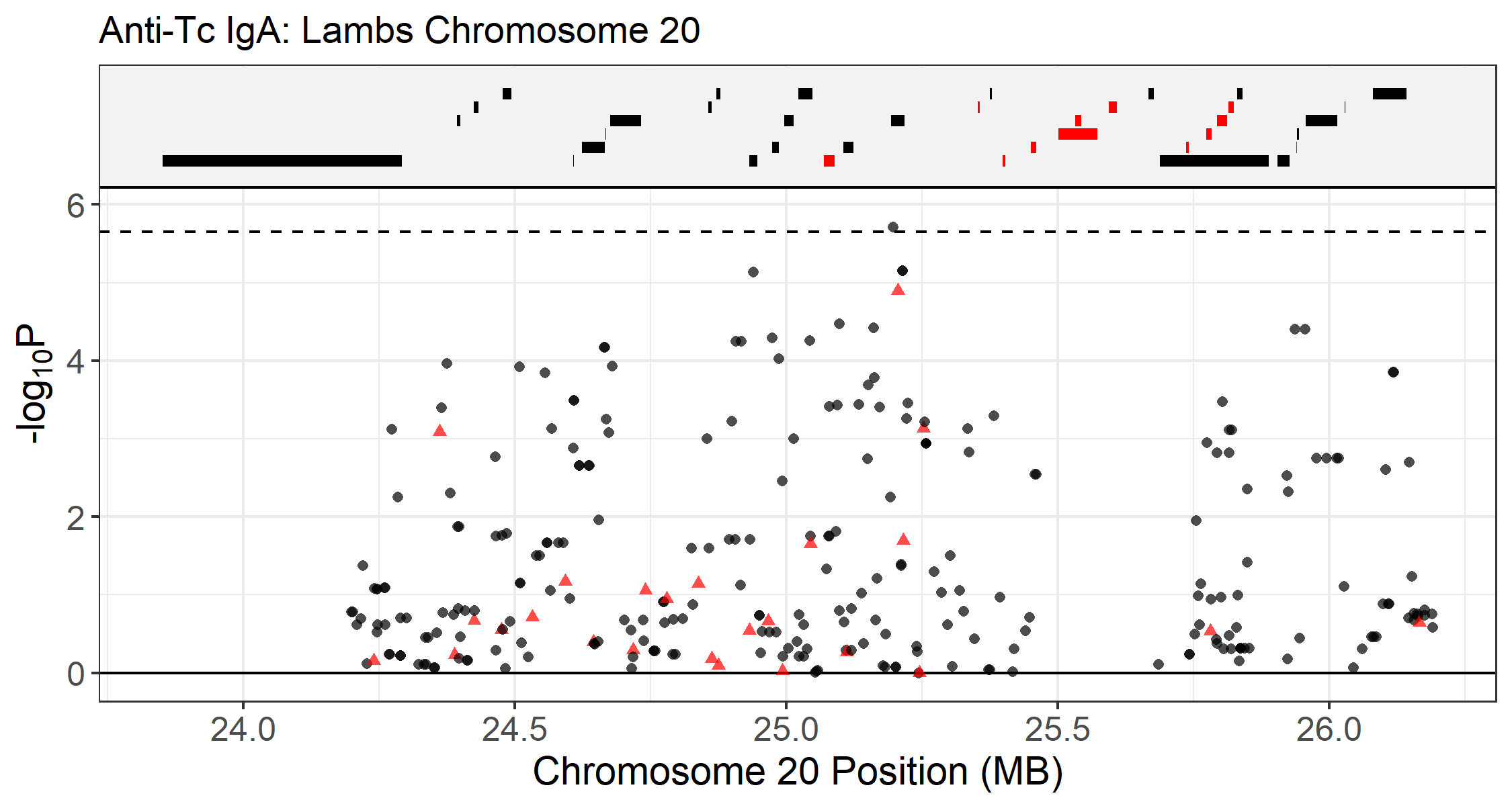

Supplement: S10 Fig — The dotted line indicates the genome-wide significance threshold equivalent to an experiment-wide threshold of P = 0.05. Points are colour-coded by their imputation status i.e. from the SNP50 chip (red triangles) or imputed from the Ovine HD chip (black points). Underlying data, sample sizes and effect sizes are provided in S6 Table. Gene positions are shown in the grey panel at the top of each plot and were obtained from Ensembl (gene build ID Oar_v3.1.94) and are provided in S7 Table. Genes coloured red have GO terms associated with immune traits (S8 Table). (TIF) [file pgen.1008461.s010.tif]

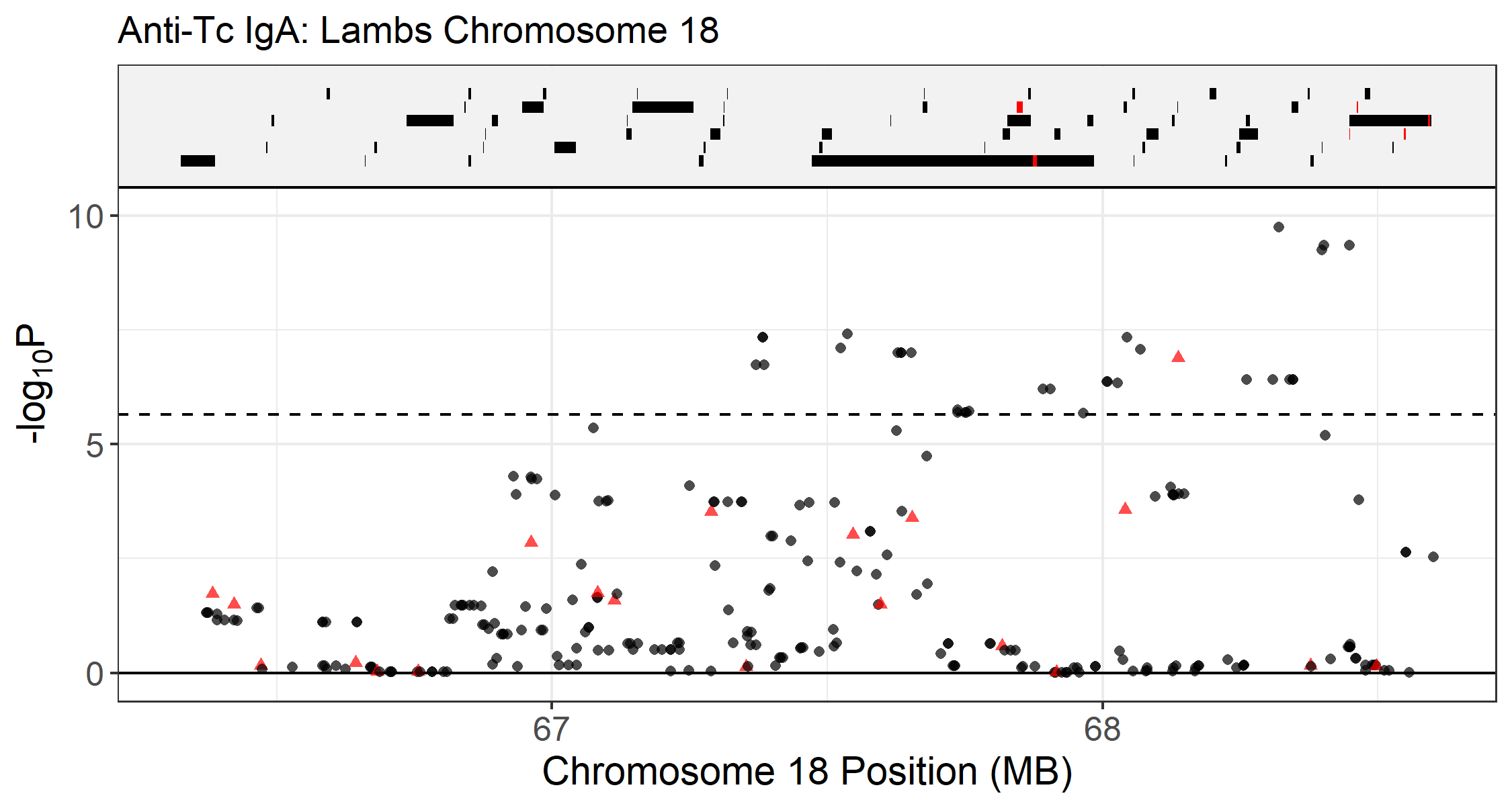

Supplement: S11 Fig — The dotted line indicates the genome-wide significance threshold equivalent to an experiment-wide threshold of P = 0.05. Points are colour-coded by their imputation status i.e. from the SNP50 chip (red triangles) or imputed from the Ovine HD chip (black points). Underlying data, sample sizes and effect sizes are provided in S6 Table. Gene positions are shown in the grey panel at the top of each plot and were obtained from Ensembl (gene build ID Oar_v3.1.94) and are provided in S7 Table. Genes coloured red have GO terms associated with immune traits (S8 Table). (TIF) [file pgen.1008461.s011.tif]

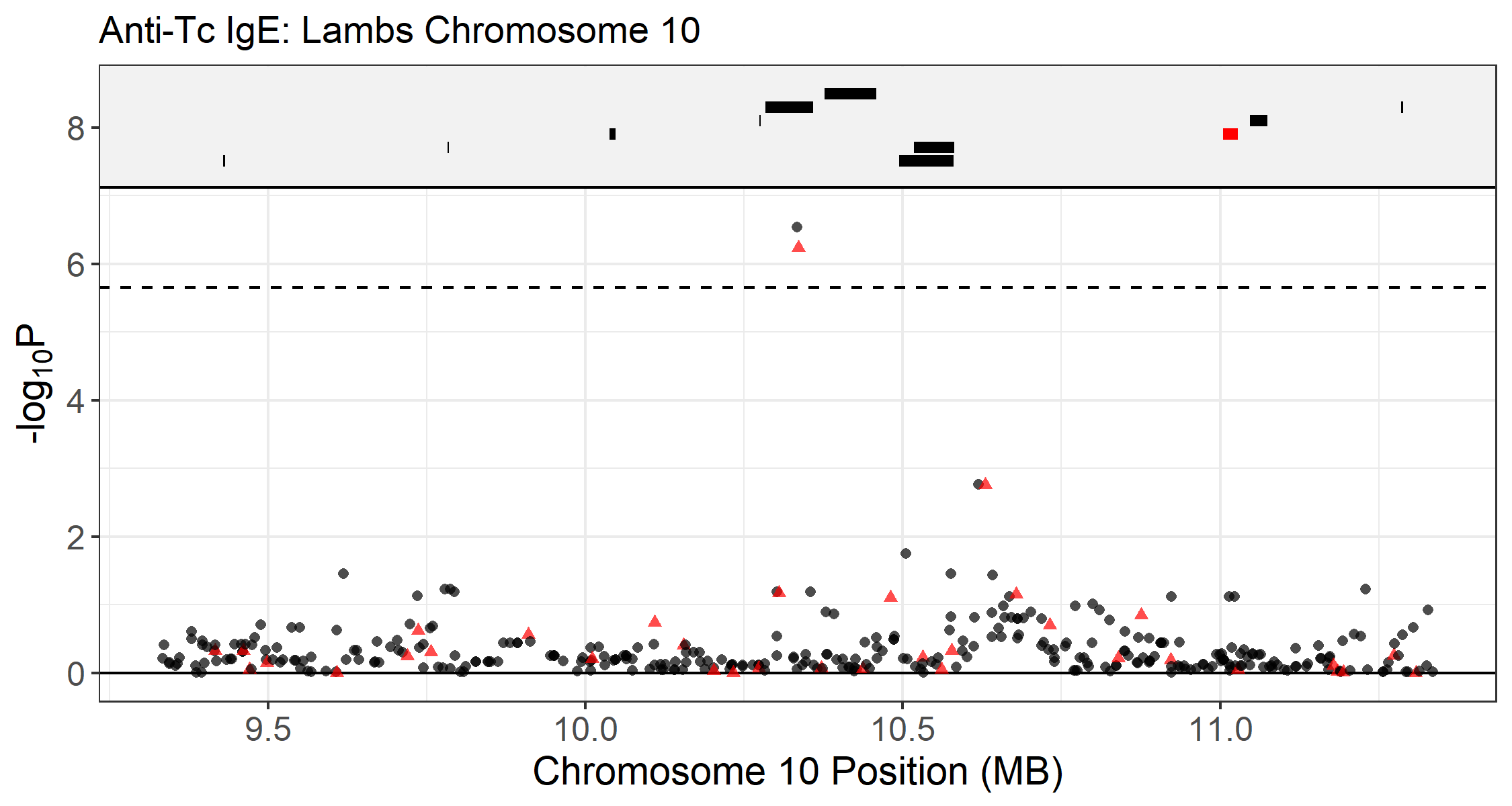

Supplement: S12 Fig — The dotted line indicates the genome-wide significance threshold equivalent to an experiment-wide threshold of P = 0.05. Points are colour-coded by their imputation status i.e. from the SNP50 chip (red triangles) or imputed from the Ovine HD chip (black points). Underlying data, sample sizes and effect sizes are provided in S6 Table. Gene positions are shown in the grey panel at the top of each plot and were obtained from Ensembl (gene build ID Oar_v3.1.94) and are provided in S7 Table. Genes coloured red have GO terms associated with immune traits (S8 Table). (TIF) [file pgen.1008461.s012.tif]

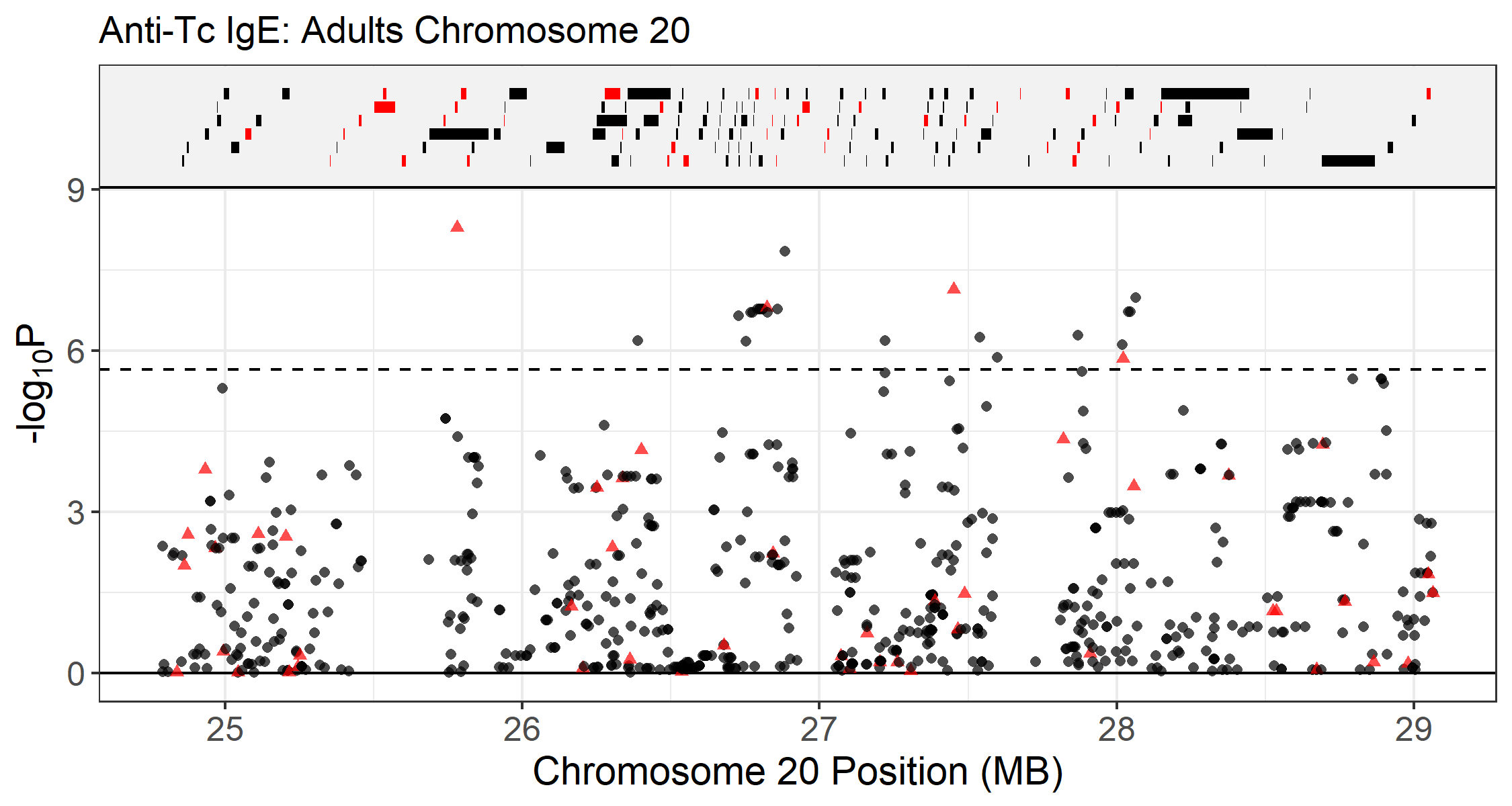

Supplement: S13 Fig — The dotted line indicates the genome-wide significance threshold equivalent to an experiment-wide threshold of P = 0.05. Points are colour-coded by their imputation status i.e. from the SNP50 chip (red triangles) or imputed from the Ovine HD chip (black points). Underlying data, sample sizes and effect sizes are provided in S6 Table. Gene positions are shown in the grey panel at the top of each plot and were obtained from Ensembl (gene build ID Oar_v3.1.94) and are provided in S7 Table. Genes coloured red have GO terms associated with immune traits (S8 Table). (TIF) [file pgen.1008461.s013.tif]

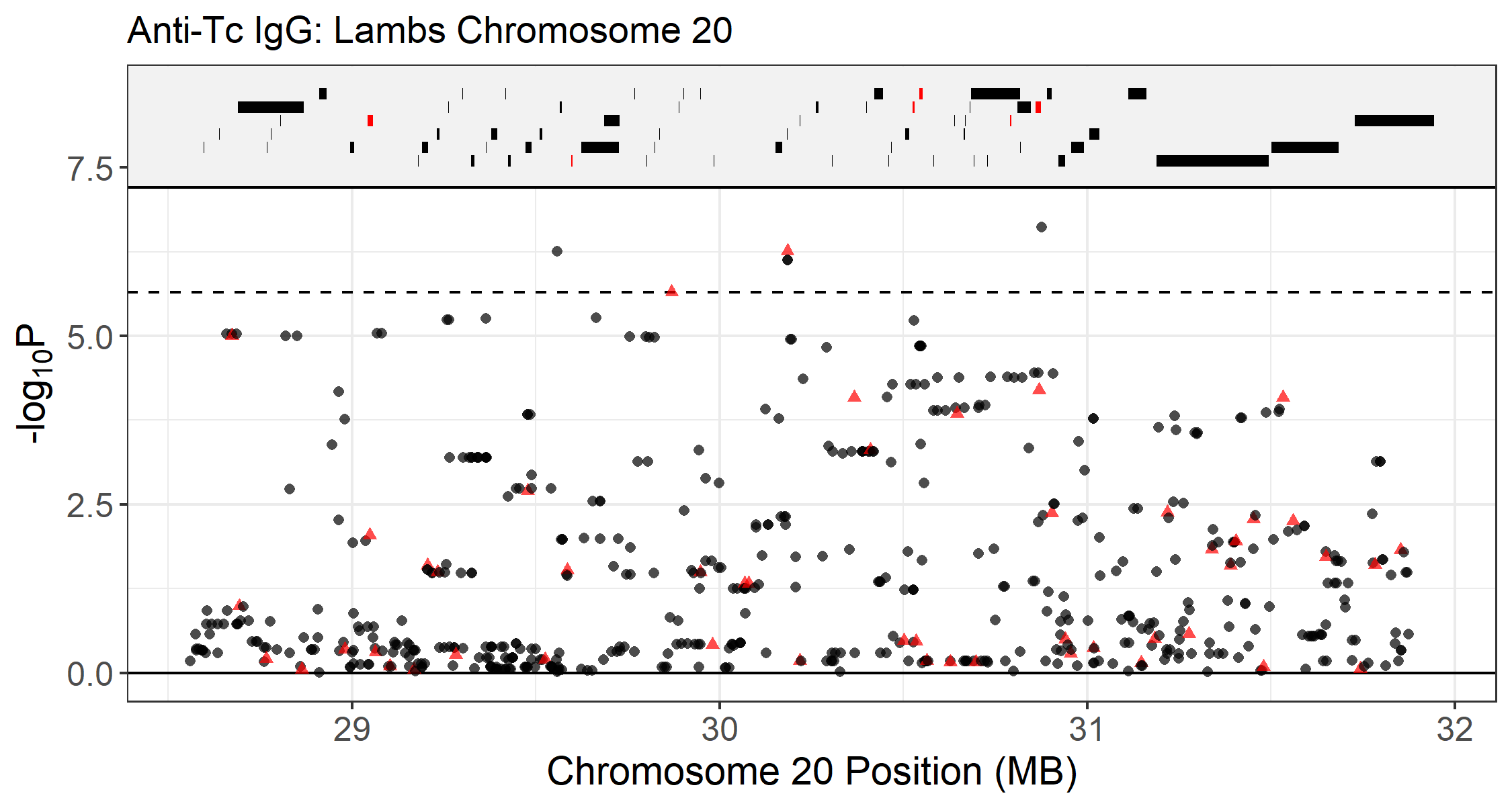

Supplement: S14 Fig — The dotted line indicates the genome-wide significance threshold equivalent to an experiment-wide threshold of P = 0.05. Points are colour-coded by their imputation status i.e. from the SNP50 chip (red triangles) or imputed from the Ovine HD chip (black points). Underlying data, sample sizes and effect sizes are provided in S6 Table. Gene positions are shown in the grey panel at the top of each plot and were obtained from Ensembl (gene build ID Oar_v3.1.94) and are provided in S7 Table. Genes coloured red have GO terms associated with immune traits (S8 Table). (TIF) [file pgen.1008461.s014.tif]

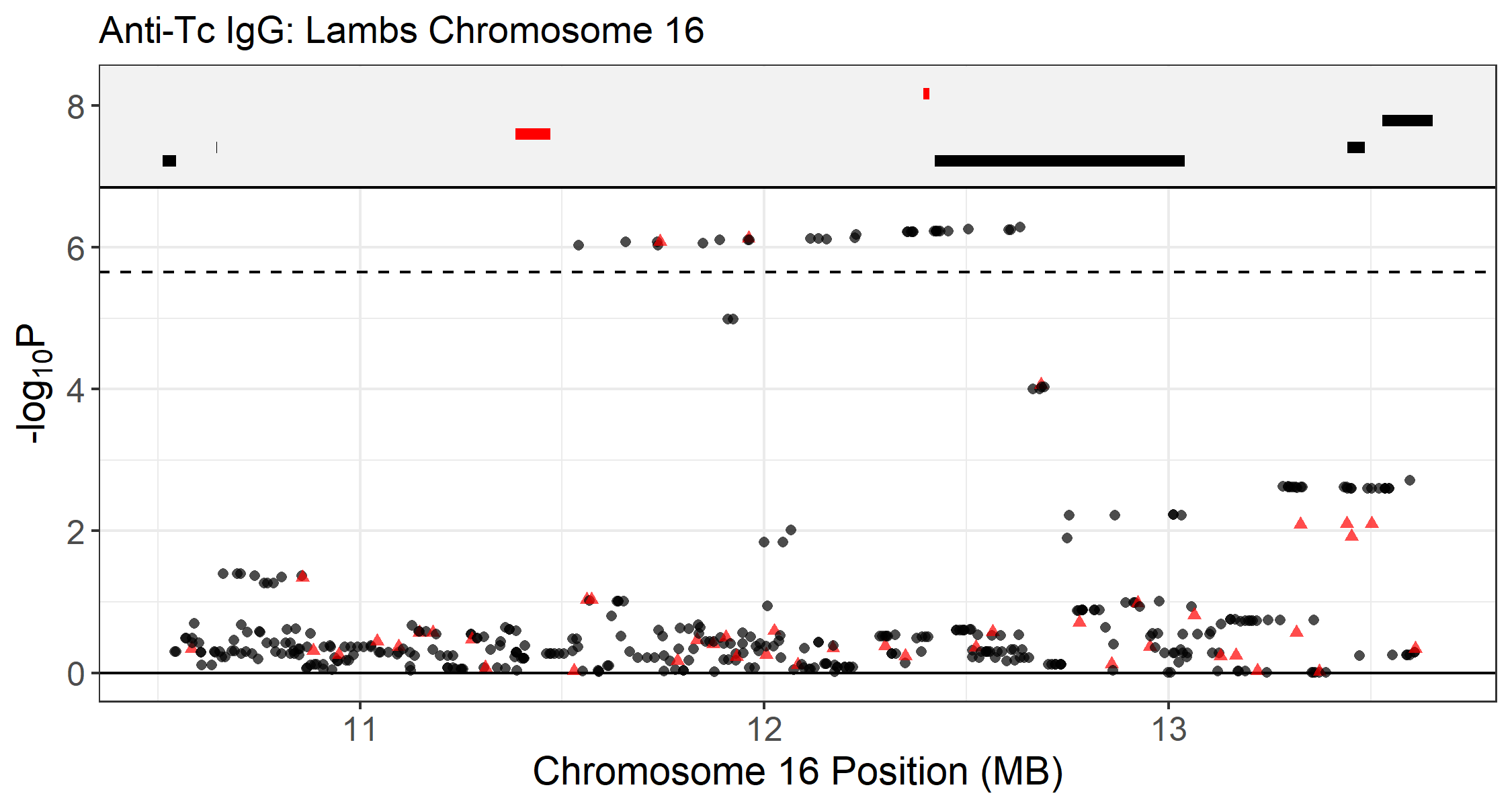

Supplement: S15 Fig — The dotted line indicates the genome-wide significance threshold equivalent to an experiment-wide threshold of P = 0.05. Points are colour-coded by their imputation status i.e. from the SNP50 chip (red triangles) or imputed from the Ovine HD chip (black points). Underlying data, sample sizes and effect sizes are provided in S6 Table. Gene positions are shown in the grey panel at the top of each plot and were obtained from Ensembl (gene build ID Oar_v3.1.94) and are provided in S7 Table. Genes coloured red have GO terms associated with immune traits (S8 Table). (TIF) [file pgen.1008461.s015.tif]
