# Supplementary material for: The genetic architecture of helminth-specific immune responses in a wild population of Soay sheep (Ovis aries)
Source: PLoS Genet. 2019 Nov 7;15(11):e1008461. doi: 10.1371/journal.pgen.1008461 (PMC6863570; doi:10.1371/journal.pgen.1008461)
Supplement: S1 Table — Slope, intercept, adjusted R2 and P-values are given for linear regressions. (DOCX) [file pgen.1008461.s016.docx]

**Table S1:** Correlations between anti-*Teladorsagia circumcincta* antibody levels in lambs and adults. Slope, intercept, adjusted R^2^ and P-values are given for linear regressions.

| Measure X | Measure Y | age | slope | intercept | Adj.R2 | P |
| --- | --- | --- | --- | --- | --- | --- |
| Anti-Tc IgA | Anti-Tc IgG | Lambs | 0.153 | 0.117 | 0.175 | 5.29E-104 |
| Anti-Tc IgE | Anti-Tc IgG | Lambs | 0.492 | 0.186 | 0.09 | 4.15E-52 |
| Anti-Tc IgE | Anti-Tc IgA | Lambs | 1.256 | 0.61 | 0.078 | 2.56E-45 |
| Anti-Tc IgA | Anti-Tc IgG | Adults | 0.04 | 0.565 | 0.01 | 1.16E-10 |
| Anti-Tc IgE | Anti-Tc IgG | Adults | 0.036 | 0.599 | 0.005 | 6.47E-06 |
| Anti-Tc IgE | Anti-Tc IgA | Adults | 0.145 | 1.4 | 0.012 | 1.28E-12 |
