# Supplementary material for: The genetic architecture of helminth-specific immune responses in a wild population of Soay sheep (Ovis aries)
Source: PLoS Genet. 2019 Nov 7;15(11):e1008461. doi: 10.1371/journal.pgen.1008461 (PMC6863570; doi:10.1371/journal.pgen.1008461)
Supplement: S2 Table — Age is the age in days during the August catch for lambs, and age in years for adults. Wald statistics are given for the significance of each effect as included in the model. (DOCX) [file pgen.1008461.s017.docx]

**Table S2.** Fixed effects results from animal models of anti-*Teladorsagia circumcincta* IgA, IgE and IgG for lambs, and adults. Age is the age in days during the August catch for lambs, and age in years for adults. Wald statistics are given for the significance of each effect as included in the model.

| Trait | Age | Effect | Effect Level | Solution | Standard Error | Z.Ratio | Wald Statistic | Wald DF | P |
| --- | --- | --- | --- | --- | --- | --- | --- | --- | --- |
| Anti-Tc IgA | Lambs | (Intercept) |  | -0.281 | 0.199 | -1.415 | 770.430 | 1 | 0 |
|  |  | Age |  | 0.009 | 0.002 | 5.387 | 29.024 | 1 | 7.15E-08 |
|  |  | Sex | F | 0.000 | NA | NA | 11.742 | 1 | 6.11 E -4 |
|  |  | Sex | M | -0.065 | 0.019 | -3.341 |  |  |  |
|  | Adults | (Intercept) |  | 1.408 | 0.027 | 52.818 | 4383.562 | 1 | 0 |
|  |  | Age |  | 0.020 | 0.003 | 6.433 | 41.379 | 1 | 1.25E-10 |
|  |  | Sex | F | 0.000 | NA | NA | 0.502 | 1 | 0.479 |
|  |  | Sex | M | -0.010 | 0.025 | -0.408 |  |  |  |
| Anti-Tc IgE | Lambs | (Intercept) |  | -0.244 | 0.048 | -5.112 | 249.859 | 1 | 0 |
|  |  | Age |  | 0.003 | 0.000 | 7.033 | 49.467 | 1 | 2.02E-12 |
|  |  | Sex | F | 0.000 | NA | NA | 0.300 | 1 | 0.584 |
|  |  | Sex | M | -0.002 | 0.005 | -0.431 |  |  |  |
|  | Adults | (Intercept) |  | 0.613 | 0.019 | 31.622 | 1974.675 | 1 | 0 |
|  |  | Age |  | 0.029 | 0.003 | 11.756 | 138.196 | 1 | 0 |
|  |  | Sex | F | 0.000 | NA | NA | 2.662 | 1 | 0.103 |
|  |  | Sex | M | -0.021 | 0.020 | -1.031 |  |  |  |
| Anti-Tc IgG | Lambs | (Intercept) |  | -0.459 | 0.078 | -5.905 | 409.237 | 1 | 0 |
|  |  | Age |  | 0.006 | 0.001 | 9.325 | 86.950 | 1 | 0 |
|  |  | Sex | F | 0.000 | NA | NA | 15.708 | 1 | 7.39E-05 |
|  |  | Sex | M | -0.029 | 0.007 | -3.837 |  |  |  |
|  | Adults | (Intercept) |  | 0.689 | 0.016 | 41.915 | 1729.272 | 1 | 0 |
|  |  | Age |  | -0.015 | 0.002 | -8.845 | 78.234 | 1 | 0 |
|  |  | Sex | F | 0.000 | NA | NA | 36.348 | 1 | 1.65E-09 |
|  |  | Sex | M | -0.066 | 0.010 | -6.502 |  |  |  |
