# Supplementary material for: The genetic architecture of helminth-specific immune responses in a wild population of Soay sheep (Ovis aries)
Source: PLoS Genet. 2019 Nov 7;15(11):e1008461. doi: 10.1371/journal.pgen.1008461 (PMC6863570; doi:10.1371/journal.pgen.1008461)
Supplement: S3 Table — Wald statistics are given for the significance of each effect as included in the model. Sample sizes are provided in Table 1. Fixed effect structures and results are provided in S2 Table. (DOCX) [file pgen.1008461.s018.docx]

**Table S3.** Random effects results from animal models of anti-*Teladorsagia circumcincta* IgA, IgE and IgG for lambs and adults. Wald statistics are given for the significance of each effect as included in the model. Sample sizes are provided in Table 1. Fixed effect structures and results are provided in Table S2.

| Trait | Age | Random Effect | Variance Component | Standard Error | Z Ratio | Effect (Proportion of Variance) | Standard Error |
| --- | --- | --- | --- | --- | --- | --- | --- |
| Anti-Tc IgA | Lambs | Birth Year | 0.0131 | 0.0052 | 2.5088 | 0.0529 | 0.0202 |
|  |  | Run Date | 0.0000 | 0.0000 | 16.2020 | 0.0000 | 0.0000 |
|  |  | Plate ID | 0.0039 | 0.0025 | 1.5831 | 0.0157 | 0.0098 |
|  |  | Mother Identity | 0.0108 | 0.0047 | 2.3049 | 0.0436 | 0.0188 |
|  |  | Additive Genetic | 0.0966 | 0.0110 | 8.7613 | 0.3890 | 0.0372 |
|  |  | Residual | 0.1239 | 0.0076 | 16.2020 | 0.4989 | 0.0366 |
|  | Adults | Capture Year | 0.0016 | 0.0011 | 1.4760 | 0.0052 | 0.0035 |
|  |  | Birth Year | 0.0031 | 0.0021 | 1.4831 | 0.0102 | 0.0068 |
|  |  | Run Date | 0.0010 | 0.0021 | 0.4773 | 0.0032 | 0.0068 |
|  |  | Plate ID | 0.0074 | 0.0023 | 3.2042 | 0.0242 | 0.0075 |
|  |  | Mother Identity | 0.0000 | 0.0000 | 34.6559 | 0.0000 | 0.0000 |
|  |  | Additive Genetic | 0.1747 | 0.0170 | 10.2746 | 0.5732 | 0.0363 |
|  |  | Perm Environment | 0.0568 | 0.0083 | 6.8239 | 0.1863 | 0.0303 |
|  |  | Residual | 0.0603 | 0.0017 | 34.6559 | 0.1977 | 0.0101 |
| Anti-Tc IgE | Lambs | Birth Year | 0.0004 | 0.0002 | 1.9569 | 0.0305 | 0.0153 |
|  |  | Run Date | 0.0000 | 0.0000 | 20.5645 | 0.0000 | 0.0000 |
|  |  | Plate ID | 0.0004 | 0.0002 | 2.1460 | 0.0288 | 0.0132 |
|  |  | Mother Identity | 0.0001 | 0.0002 | 0.3847 | 0.0067 | 0.0174 |
|  |  | Additive Genetic | 0.0029 | 0.0005 | 5.9041 | 0.2122 | 0.0334 |
|  |  | Residual | 0.0097 | 0.0005 | 20.5645 | 0.7219 | 0.0360 |
|  | Adults | Capture Year | 0.0023 | 0.0010 | 2.3497 | 0.0134 | 0.0057 |
|  |  | Birth Year | 0.0000 | 0.0000 | 34.8017 | 0.0000 | 0.0000 |
|  |  | Run Date | 0.0000 | 0.0000 | 34.8017 | 0.0000 | 0.0000 |
|  |  | Plate ID | 0.0036 | 0.0010 | 3.7558 | 0.0208 | 0.0055 |
|  |  | Mother Identity | 0.0008 | 0.0032 | 0.2559 | 0.0047 | 0.0182 |
|  |  | Additive Genetic | 0.0811 | 0.0091 | 8.9316 | 0.4662 | 0.0385 |
|  |  | Perm Environment | 0.0440 | 0.0059 | 7.4223 | 0.2531 | 0.0368 |
|  |  | Residual | 0.0420 | 0.0012 | 34.8017 | 0.2418 | 0.0117 |
| Anti-Tc IgG | Lambs | Birth Year | 0.0025 | 0.0010 | 2.4917 | 0.0703 | 0.0266 |
|  |  | Run Date | 0.0000 | 0.0000 | 18.4285 | 0.0000 | 0.0000 |
|  |  | Plate ID | 0.0015 | 0.0006 | 2.5077 | 0.0411 | 0.0161 |
|  |  | Mother Identity | 0.0007 | 0.0007 | 1.0990 | 0.0203 | 0.0184 |
|  |  | Additive Genetic | 0.0097 | 0.0013 | 7.2460 | 0.2739 | 0.0344 |
|  |  | Residual | 0.0210 | 0.0011 | 18.4285 | 0.5944 | 0.0381 |
|  | Adults | Capture Year | 0.0008 | 0.0005 | 1.7634 | 0.0180 | 0.0101 |
|  |  | Birth Year | 0.0001 | 0.0002 | 0.5680 | 0.0027 | 0.0048 |
|  |  | Run Date | 0.0038 | 0.0015 | 2.5122 | 0.0803 | 0.0300 |
|  |  | Plate ID | 0.0029 | 0.0008 | 3.5223 | 0.0618 | 0.0172 |
|  |  | Mother Identity | 0.0000 | 0.0000 | 34.6355 | 0.0000 | 0.0000 |
|  |  | Additive Genetic | 0.0110 | 0.0017 | 6.4888 | 0.2347 | 0.0330 |
|  |  | Perm Environment | 0.0134 | 0.0013 | 10.0241 | 0.2854 | 0.0296 |
|  |  | Residual | 0.0149 | 0.0004 | 34.6355 | 0.3172 | 0.0159 |
