# Supplementary material for: The genetic architecture of helminth-specific immune responses in a wild population of Soay sheep (Ovis aries)
Source: PLoS Genet. 2019 Nov 7;15(11):e1008461. doi: 10.1371/journal.pgen.1008461 (PMC6863570; doi:10.1371/journal.pgen.1008461)
Supplement: S4 Table — Results are from a linear regression with t+1 levels as the response variable. (DOCX) [file pgen.1008461.s019.docx]

**Table S4.** Temporal correlations in anti-*Teladorsagia circumcincta* IgA, IgE and IgG levels at time t and t+1 (in years) as shown in Figure S4. Results are from a linear regression with t+1 levels as the response variable.

| Model | Slope | Intercept | Adjusted R2 | P |
| --- | --- | --- | --- | --- |
| Anti-Tc IgA | 0.801 | 0.318 | 0.656 | 0.00E+00 |
| Anti-Tc IgE | 0.848 | 0.154 | 0.69 | 0.00E+00 |
| Anti-Tc IgG | 0.519 | 0.287 | 0.293 | 2.30E-146 |
