# Supplementary material for: The genetic architecture of helminth-specific immune responses in a wild population of Soay sheep (Ovis aries)
Source: PLoS Genet. 2019 Nov 7;15(11):e1008461. doi: 10.1371/journal.pgen.1008461 (PMC6863570; doi:10.1371/journal.pgen.1008461)
Supplement: S9 Table — These results are visualized in S6 Fig. Associated Wald statistics and P values are provided in Tables 2, S5 and S6. (DOCX) [file pgen.1008461.s024.docx]

**Table S9:** Genotypic effects at the most significant GWAS loci (Table 2). These results are visualized in Figure S6. Associated Wald statistics and P values are provided in Tables 2, S5 and S6.

| Trait | Age | SNP Locus | Genotype | Solution | Standard Error | Z |
| --- | --- | --- | --- | --- | --- | --- |
|  |  |  |  |  |  | Ratio |
| IgA | Lambs | s03219.1 | A/A | 0.000 | 0.000 | NA |
|  |  |  | A/G | 0.091 | 0.023 | 3.956 |
|  |  |  | G/G | 0.202 | 0.038 | 5.343 |
|  |  | oar3_OAR20_25196550 | A/A | 0.000 | 0.000 | NA |
|  |  |  | A/G | -0.083 | 0.027 | -3.111 |
|  |  |  | G/G | -0.175 | 0.034 | -5.114 |
|  |  | oar3_OAR24_10616039 | A/A | 0.000 | 0.000 | NA |
|  |  |  | A/G | -0.383 | 0.029 | -13.160 |
|  |  |  | G/G | -0.718 | 0.040 | -17.835 |
|  | Adults | oar3_OAR24_10858856 | A/A | 0.000 | 0.000 | NA |
|  |  |  | A/G | -0.192 | 0.025 | -7.781 |
|  |  |  | G/G | -0.424 | 0.032 | -13.278 |
| IgE | Lambs | oar3_OAR10_10333145 | A/A | 0.000 | 0.000 | NA |
|  |  |  | A/G | 2.819 | 0.550 | 5.128 |
|  |  |  | G/G | 2.929 | 0.548 | 5.349 |
|  | Adults | OAR20_27259292.1 | A/A | 0.000 | 0.000 | NA |
|  |  |  | A/G | 0.150 | 0.030 | 4.954 |
|  |  |  | G/G | 0.211 | 0.036 | 5.904 |
| IgG | Lambs | oar3_OAR16_12632988 | A/A | 0.000 | 0.000 | NA |
|  |  |  | A/G | 0.026 | 0.018 | 1.448 |
|  |  |  | G/G | 0.529 | 0.100 | 5.268 |
|  |  | oar3_OAR20_30876754 | A/A | 0.000 | 0.000 | NA |
|  |  |  | A/G | -0.072 | 0.021 | -3.488 |
|  |  |  | G/G | -0.104 | 0.021 | -4.938 |
