# Supplementary material for: The genetic architecture of helminth-specific immune responses in a wild population of Soay sheep (Ovis aries)
Source: PLoS Genet. 2019 Nov 7;15(11):e1008461. doi: 10.1371/journal.pgen.1008461 (PMC6863570; doi:10.1371/journal.pgen.1008461)
Supplement: S10 Table — The Wald statistic is given for the sex by genotype interaction term. These results are visualized in S7 Fig. (DOCX) [file pgen.1008461.s025.docx]

**Table S10:** Sex-specific SNP effects at the most significant GWAS loci (Table 2). The Wald statistic is given for the sex by genotype interaction term. These results are visualized in Figure S7.

| Trait | Age | SNP Locus | Sex | Genotype | Solution | Standard Error | Z  Ratio | Wald  Statistic |
| --- | --- | --- | --- | --- | --- | --- | --- | --- |
| IgA | Lambs | s03219.1 | F | A/A | 0.000 | 0.000 | NA | 42.76 |
|  |  |  |  | A/G | -0.108 | 0.049 | -2.190 | df = 5 |
|  |  |  |  | G/G | -0.182 | 0.052 | -3.533 | P = 4.13E-08 |
|  |  |  | M | A/A | -0.051 | 0.060 | -0.847 |  |
|  |  |  |  | A/G | -0.156 | 0.049 | -3.178 |  |
|  |  |  |  | G/G | -0.270 | 0.052 | -5.193 |  |
|  |  | oar3_OAR20_25196550 | F | A/A | 0.000 | 0.000 | NA | 36.88 |
|  |  |  |  | A/G | -0.107 | 0.035 | -3.037 | df = 5 |
|  |  |  |  | G/G | -0.204 | 0.044 | -4.668 | P = 6.33E-07 |
|  |  |  | M | A/A | -0.103 | 0.041 | -2.525 |  |
|  |  |  |  | A/G | -0.158 | 0.036 | -4.382 |  |
|  |  |  |  | G/G | -0.246 | 0.044 | -5.611 |  |
|  |  | oar3_OAR24_10616039 | F | A/A | 0.000 | 0.000 | NA | 190.2 |
|  |  |  |  | A/G | -0.227 | 0.033 | -6.890 | df = 5 |
|  |  |  |  | G/G | -0.458 | 0.042 | -10.988 | P = 0 |
|  |  |  | M | A/A | -0.108 | 0.037 | -2.953 |  |
|  |  |  |  | A/G | -0.262 | 0.034 | -7.763 |  |
|  |  |  |  | G/G | -0.496 | 0.041 | -12.138 |  |
|  | Adults | oar3_OAR24_10858856 | F | A/A | 0.000 | 0.000 | NA | 325.7 |
|  |  |  |  | A/G | -0.400 | 0.034 | -11.683 | df = 5 |
|  |  |  |  | G/G | -0.751 | 0.046 | -16.295 | P = 0 |
|  |  |  | M | A/A | -0.054 | 0.046 | -1.167 |  |
|  |  |  |  | A/G | -0.402 | 0.040 | -10.053 |  |
|  |  |  |  | G/G | -0.706 | 0.055 | -12.774 |  |
| IgE | Lambs | oar3_OAR10_10333145 | F | A/G | 0.000 | 0.000 | NA | 3.061 |
|  |  |  |  | G/G | 0.059 | 0.101 | 0.586 | df = 3 |
|  |  |  | M | A/G | -0.117 | 0.134 | -0.875 | P = 0.382 |
|  |  |  |  | G/G | 0.048 | 0.102 | 0.475 |  |
|  | Adults | OAR20_27259292.1 | F | A/A | 0.000 | 0.000 | NA | 41.31 |
|  |  |  |  | A/G | -0.052 | 0.027 | -1.958 | df = 5 |
|  |  |  |  | G/G | -0.233 | 0.042 | -5.508 | P = 8.10E-08 |
|  |  |  | M | A/A | -0.016 | 0.034 | -0.466 |  |
|  |  |  |  | A/G | -0.096 | 0.032 | -3.021 |  |
|  |  |  |  | G/G | -0.217 | 0.051 | -4.278 |  |
| IgG | Lambs | oar3_OAR16_12632988 | F | A/A | 0.000 | 0.000 | NA | 13.94 |
|  |  |  |  | A/G | 0.017 | 0.023 | 0.713 | df = 3 |
|  |  |  | M | A/A | -0.027 | 0.008 | -3.432 | P = 2.99E-3 |
|  |  |  |  | A/G | 0.008 | 0.025 | 0.320 |  |
|  |  | oar3_OAR20_30876754 | F | A/A | 0.000 | 0.000 | NA | 47.70 |
|  |  |  |  | A/G | -0.033 | 0.029 | -1.133 | df = 5 |
|  |  |  |  | G/G | -0.078 | 0.029 | -2.654 | P = 4.09E-9 |
|  |  |  | M | A/A | 0.029 | 0.038 | 0.766 |  |
|  |  |  |  | A/G | -0.080 | 0.029 | -2.720 |  |
|  |  |  |  | G/G | -0.099 | 0.029 | -3.362 |  |
